# The Model of the Extraction Process of Rare Metals Under Condition of Filtration Combustion Wave

**DOI:** 10.3389/fchem.2020.511502

**Published:** 2020-11-05

**Authors:** Eugene A. Salgansky, Nickolay A. Lutsenko, Mario Toledo

**Affiliations:** ^1^Institute of Problems of Chemical Physics, Russian Academy of Sciences, Chernogolovka, Russia; ^2^Institute of Automation and Control Processes Far East Branch of the Russian Academy of Sciences, Vladivostok, Russia; ^3^Far Eastern Federal University, Vladivostok, Russia; ^4^Department of Mechanical Engineering, Universidad Técnica Federico Santa Maria, Valparaíso, Chile

**Keywords:** rare and precious metals, mass transfer, filtration combustion, concentration, extraction, modeling

## Abstract

To study the mass transfer of metal compounds, a model of filtration combustion of metal-containing combustible mixtures is developed. Using cadmium-containing mixture as an example, the main characteristics of filtration combustion are determined when the gas pressure at the reactor inlet is constant. It is shown that under the conditions of a filtration combustion wave, a metal can evaporate into the gas phase and be transferred with gas through the reactor. Due to the evaporation and condensation of cadmium, it is transported and accumulated before the combustion front. The possibility of controlling the mass transfer of metal compounds under the conditions of a filtration combustion wave with the aim of concentrating them is shown. It is revealed that a 4-fold increase in the pressure difference at the open boundaries of the reactor can lead to a decrease in the maximum metal concentration by about 1.5 times. An increase in the concentration of metals due to mass transfer will subsequently make it economically feasible to extract them by traditional methods.

## Introduction

Rare metals have actively entered the scope of industrial applications: electronics, aviation, superconductors, mobile power sources, etc. (Frohlich et al., 2017). As a rule, they are not widespread in the earth's crust; therefore, the volume of production and use of rare metals is relatively small due to the high degree of dispersion and complexity of extraction methods. However, without the use of rare and precious metals, the sustainable development of new technologies and the production of materials with necessary properties are impossible. Rare metals do not have their own large deposits and are associated elements of the ore layers (Jowitt et al., 2018). With the development of more accurate analysis methods and more efficient extraction technologies, the raw material base of rare and valuable metals began to expand. Currently, waste from metallurgical industries (Johnson, 2014), waste electronics (Sthiannopkao and Wong, 2013), as well as coal and oil are considered as an alternative raw material base for rare and precious metals. The content of some metals in coal and oil can reach several hundred grams per ton, and in their ash residue it can reach several kilograms per ton (Dai and Finkelman, 2017; Salgansky et al., 2019).

The main technologies for the extraction of rare metals are the following: metallurgical methods (Jha et al., 2001), extraction methods (Kuang and Liao, 2018), methods of mechanical separation (Frohlich et al., 2017). Biological methods for the extraction of rare and precious metals are being actively developed: biohydrometallurgical (Erüst et al., 2013), bioelectrochemical (Nancharaiah et al., 2016), bioextraction (Watling et al., 2010). As a rule, these methods are used for the extraction of metals from ores. As for the metals contained in oils and coals, it is rational to remove the organics (for example, by burning) and leave the metals in the ash residue to extract them. In this case, due to the low ash content of oils and coals, the concentration of metals in the ash will significantly increase. The method of filtration combustion with super-adiabatic heating is a way of efficiently burning of various combustible materials (Manelis et al., 2011; Toledo et al., 2018). The possibility of concentrating and separation of molybdenum compounds (Manelis et al., 2016), zinc (Rozenberg et al., 2009) in a laboratory reactor has been experimentally shown. The theory of filtration combustion of solid fuels is well developed (Aldushin, 1997). Mathematical models have been developed that describe the filtration combustion of solid fuels both in the one-dimensional (Salganskii et al., 2003; Lutsenko, 2013) and two-dimensional approaches (Levin and Lutsenko, 2017; Lutsenko, 2018). However, up to date, the models of filtration combustion of solid fuels have not taken into account the presence of metal compounds in porous combustible media.

The aim of this work is to develop a mathematical model that allows studying the behavior of metal compounds under conditions of a filtration combustion wave. The proposed model is a development of the one described in (Lutsenko and Salgansky, 2019).

## Mathematical Model and Numerical Method

**Figure 1:**
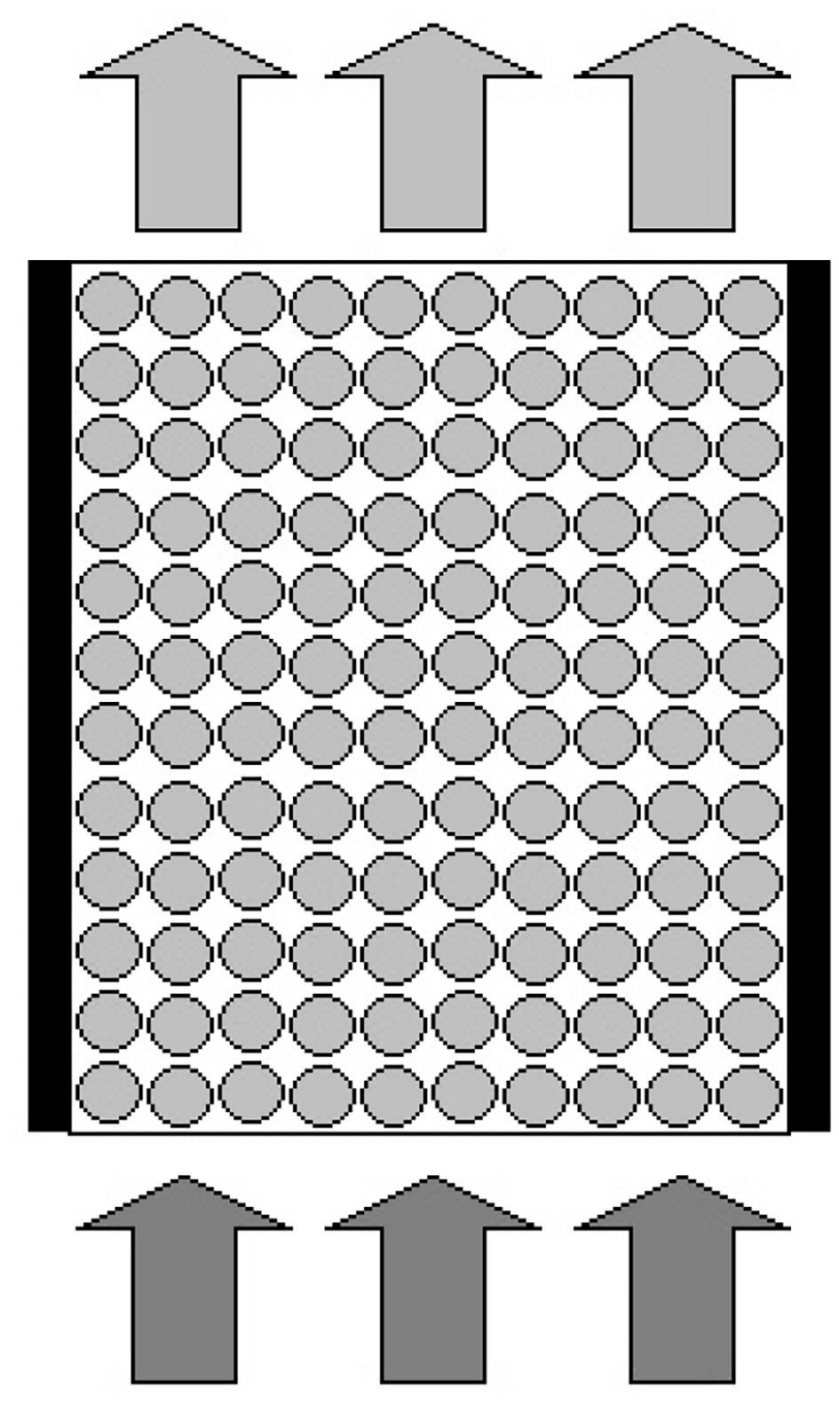
Sketch of the porous object.

Consider vertical packed bed reactor, which has impermeable side walls and is opened to an atmosphere at a bottom and at a top. Gas is pumped through the lower open boundary (inlet) of the object, passes through the porous medium and flows through the upper boundary (outlet) (Figure 1).

Suppose that a condensed porous matter consists of combustible component (fuel), inert component, solid products of reaction and metal-containing substance. The gas phase can include evaporated metal. The solid fuel transforms into gaseous and solid products in the reaction with gaseous oxidizer, so we have the following expression:

(1) $$\begin{array}{l} \text{Solid fuel}+\;\left(\mu_{g}\right)\text{Oxidizer}\to \left(1+\mu_{g}-\mu_{p}\right)\text{Gaseous product}\\ \;+\;\left(\mu_{p}\right)\text{Solid product}, \end{array}$$

where μ_*g*_ and μ_*p*_ are the mass stoichiometric coefficients for gaseous oxidizer and solid products, respectively.

Suppose that only a solid combustible component and an oxidizing agent from the gas phase (oxygen) are chemically reacted. The metal-containing substance does not chemically react and undergoes two phase transitions: when heated, it can melt and then evaporate with transforming to gaseous products. The evaporated metal can condense when the gas phase is cooled. The liquid phase of the metal remains immovable and associated with the solid (due to adsorption); when it cools, it solidifies. We assume that the melting and solidification of the metal occur at a fixed temperature *T*_*melt*_, the evaporation and condensation of the metal also occur at a fixed temperature *T*_*evap*_, and the phase transitions occur so quickly that their rates are completely determined by the heat fluxes.

The mathematical model is based on the methods of multicomponent continuum mechanics (Nigmatulin, 1990) using classical approaches of the theory of filtration combustion (Aldushin, 1997). To describe the phase transitions, we introduce the following notation: *F* – is the fraction of the liquid phase in the metal, ρ′_*cm*_ is the decrease in the effective density of the condensed metal due to evaporation (in average: mass of the condensed metal per unit volume that evaporates), ρ′_*gm*_ is the decrease in the effective density of the evaporated metal due to condensation (in average: mass of evaporated metal per unit volume that condenses). So, we can write the following mathematical model of the investigated process:

Energy equation for condensed phase:

(2) $$\begin{array}{l} \;\left(\rho_{cf}c_{cf}+\rho_{ci}c_{ci}+\rho_{cp}c_{cp}+\rho_{cm}c_{cm}\right)\frac{\partial T_{c}}{\partial t}=\\ =-\alpha \left(T_{c}-T_{g}\right)+\rho_{cf0}Q_{comb}W+\left(1-a_{g}\right)\lambda_{c}\Delta T_{c}\\ -\;\rho_{cm}Q_{melt}\frac{\partial F}{\partial t}-Q_{evap}\frac{\partial {\rho^{\prime }}_{cm}}{\partial t}, \end{array}$$

Energy equation for gas phase:

(3) $$\begin{array}{l} \rho_{g}c_{g}\left(\frac{\partial T_{g}}{\partial t}+\left(\nu_{g}\cdot \nabla \right)\;T_{g}\right)=\alpha \left(T_{c}-T_{g}\right)+a_{g}\lambda_{g}\Delta T_{g}\\ \;+\;Q_{evap}\frac{\partial {\rho^{\prime }}_{gm}}{\partial t}, \end{array}$$

Equations of continuity and state for gas:

(4) $$\begin{array}{l} \frac{\partial \rho_{g}}{\partial t}+\nabla \cdot \left(\rho_{g}\nu_{g}\right)=\left(1-\mu_{p}\right)\rho_{cf0}W+\frac{\partial {\rho^{\prime }}_{cm}}{\partial t}-\frac{\partial {\rho^{\prime }}_{gm}}{\partial t},\\ \;p=\frac{\rho_{g}RT_{g}}{a_{g}M}, \end{array}$$

Momentum conservation equation for gas:

(5) $$\begin{array}{l} \rho_{g}\left(1+\chi \left(1-a_{g}\right)\right)\;\left(\frac{\partial \nu_{g}}{\partial t}+\left(\nu_{g}\cdot \nabla \right)\;\nu_{g}\right)=-a_{g}\nabla p\\ +\;\rho_{g}g-a_{g}^{2}\frac{\mu }{k_{1}}\nu_{g}-\\ \;-\;\left[\left(1-\mu_{p}\right)\rho_{cf0}W+\;\frac{\partial {\rho^{\prime }}_{cm}}{\partial t}-\frac{\partial {\rho^{\prime }}_{gm}}{\partial t}\;\right]\nu_{g}, \end{array}$$

Equation for oxidizer concentration:

(6) $$\begin{array}{l} \rho_{g}\left(\frac{\partial C}{\partial t}+\left(\nu_{g}\cdot \nabla \right)\;C\right)=\nabla \cdot \left(\rho_{g}D_{g}\nabla C\right)-\mu_{g}\rho_{cf0}W-\\ \;-\left[\;\left(1-\mu_{p}\right)\rho_{cf0}W+\;\frac{\partial {\rho^{\prime }}_{cm}}{\partial t}-\;\frac{\partial {\rho^{\prime }}_{gm}}{\partial t}\right]C, \end{array}$$

Equations of chemical kinetics:

(7) $$\begin{array}{l} W=\left(1-\eta \right)\;C\;k\;\exp\left(-E/\left(R\;T_{c}\right)\right),\frac{\partial \eta }{\partial t}=W, \end{array}$$

Equation of mass conservation for evaporated metal in gas:

(8) $$\frac{\partial \rho_{gm}}{\partial t}+\nabla \left(\rho_{gm}\nu_{g}\right)=-\;\frac{\partial {\rho^{\prime }}_{gm}}{\partial t}+\;\frac{\partial {\rho^{\prime }}_{cm}}{\partial t}$$

Equations of mass conservation for components of condensed phase:

(9) $$\begin{array}{l} \rho_{cf}=\left(1-\eta \right)\rho_{cf0},\rho_{cp}=\mu_{p}\rho_{cf0}\eta ,\frac{\partial \rho_{cm}}{\partial t}=\\ \;-\;\frac{\partial {\rho^{\prime }}_{cm}}{\partial t}+\frac{\partial {\rho^{\prime }}_{gm}}{\partial t}, \end{array}$$

Equations for porosity:

(10) $$\begin{array}{l} a_{g}=a_{g0}+\left(a_{cf0}-a_{cpEnd}\right)\eta +\frac{\rho_{cm0}-\rho_{cm}}{\rho_{cm0}}a_{cm0}, \end{array}$$

Equations for oxidizer diffusion and gas viscosity:

(11) $$\begin{array}{l} D_{g}=D_{g0}{\left(T_{g}/273\right)}^{b},\mu =c_{s1}\frac{T_{g}^{1.5}}{c_{s2}+T_{g}}. \end{array}$$

Here *a* is the volume concentration, *b* is the exponent in the expression for diffusion coefficient, *C* is the mass concentration of oxidizer, *c* is the specific heat capacity, *c*_*s*1_ and *c*_*s*2_ are the constants in Sutherland's formula, *D*_*g*_ is the diffusion coefficient of gas, *E* is the activation energy, *g* is the gravity acceleration, *k* is the pre-exponential factor in the expression for the rate of reaction, *k*_1_ is the permeability coefficient, *M* is the molar mass of gas, *p* is the gas pressure, *Q* is the heat release, *R* is the universal gas constant, *t* is the time, *T* is the temperature, *v* is the velocity, *W* is the rate of chemical reaction, α is the constant determining the interphase heat transfer intensity, η is the degree of conversion of the solid combustible component, λ is the thermal conductivity (including radiation heat transfer according to diffusion approximation), μ is the dynamic viscosity of gas, ρ is the effective (or bulk) density (i.e., the product of phase density and volume concentration), χ is the coefficient, taking into account the inertial interaction of the phases in their relative motion (Nigmatulin, 1990), ∇ is the del (nabla) operator, Δ is the Laplace operator; subscripts: “0” denotes the initial moment, “*c*” denotes the condensed phase, “*comb*” denotes the combustion, “*End*” denotes the end point of time, “*evap*” denotes the evaporation, “*f* ” denotes the combustible component (fuel), “*g*” denotes the gas, “*i*" denotes the inert component, “*m*” denotes the metal substance, “*melt*” denotes the melting, “*p*” denotes the product of reaction.

We will use dimensionless quantities and dimensionless similarity parameters; therefore, we introduce dimensionless variables as follows: $\tilde{x}=x/H$, $\tilde{t}=t/t_{*}$, ${\tilde{v}}_{g}=v_{g}/{ṽ}_{*}$, where *H* is the characteristic size of the simulated reactor (unless otherwise specified, this is its height), t_*_ are v_*_ are the characteristic values of time and gas velocity, *x* is the Eulerian coordinate; $\tilde{p}=p/p_{*}$, ${\tilde{\rho }}_{g}=\rho_{g}/\rho_{*}$, ${\tilde{T}}_{c}=T_{c}/T_{*}$, ${\tilde{T}}_{g}=T_{g}/T_{*}$, where p_*_, ρ_*_, T_*_ are the pressure, density and temperature of the gas under “normal” conditions; ${\tilde{\rho }}_{cf}=\rho_{cf}/\rho_{sf0}$; ${\tilde{\rho }}_{cp}=\rho_{cp}/\rho_{cf0}$; $\tilde{W}=W/k$; ${\tilde{D}}_{g}={\left(T_{*}/273\right)}^{-b}D_{g}/D_{g0}$; ${\tilde{\rho }}_{cm}=\rho_{cm}/\rho_{cf0}$; ${\tilde{\rho }}_{gm}=\rho_{gm}/\rho_{*}$; $\frac{\partial {{\tilde{\rho }}^{\prime }}_{cm}}{\partial \tilde{t}}=\frac{\partial \rho \prime_{cm}}{\partial t}\frac{t_{*}}{\rho_{sf0}}$; $\frac{\partial {{\tilde{\rho }}^{\prime }}_{gm}}{\partial \tilde{t}}=\frac{\partial \rho \prime_{gm}}{\partial t}\frac{t_{*}}{\rho_{*}}$.

The following similarity parameters will be used:

$$\begin{array}{l} \text{Sh}=\frac{v_{*}t_{*}}{H},\text{S}{\text{t}}_{1}=\frac{\alpha H}{\rho_{cf0}c_{cf}v_{*}},\text{S}{\text{t}}_{2}=\frac{\alpha H}{\rho_{*}c_{g}v_{*}},\\ \text{Eu}=\frac{p_{*}}{\rho_{*}v_{*}^{2}},\text{Re}=\;\frac{\rho_{*}v_{*}H}{c_{s1}\sqrt{T_{*}}},\\ \text{Fr}=\frac{v_{*}^{2}}{gH},\text{P}{\text{e}}_{1}=\frac{v_{*}\rho_{cf0}c_{cf}H}{\lambda_{c}},\text{P}{\text{e}}_{2}=\frac{v_{*}\rho_{*}c_{g}H}{\lambda_{g}},\\ \text{Sc}=\frac{c_{s1}\sqrt{T_{*}}}{\rho_{*}D_{g0}{\left(T_{*}/273\right)}^{b}},{\tilde{Q}}_{comb}=\;\frac{Q_{comb}}{c_{cf}T_{*}},\\ Ẽ=\frac{E}{RT_{*}},\pi =\frac{k_{1}}{H^{2}},\pi_{1}=\frac{\rho_{ci}c_{ci}}{\rho_{cf0}c_{cf}},\pi_{2}=k\;t_{*},\pi_{3}\\ \;=\frac{\rho_{cf0}}{\rho_{*}},\pi_{4}=\frac{c_{cp}}{c_{cf}},{\overset{⌢}{c}}_{s2}=\;\frac{c_{s2}}{T_{*}},\\ \pi_{5}=\frac{c_{cm}}{c_{cf}},\pi_{6}=\frac{\rho_{cf0}}{\rho_{cmIst}},{\tilde{Q}}_{melt}=\frac{Q_{melt}}{c_{cf}T_{*}},{\tilde{Q}}_{evap1}\\ \;=\frac{Q_{evap}}{c_{cf}T_{*}},{\tilde{Q}}_{evap2}=\;\frac{Q_{evap}}{c_{g}T_{*}}. \end{array}$$

We can rewrite Equations (2)–(11) in dimensionless variables omitting the tilde sign:

(12) $$\begin{array}{l} \;\left(\rho_{sf}+\pi_{1}+\pi_{4}\rho_{cp}+\pi_{5}\rho_{cm}\right)\frac{\partial T_{c}}{\partial t}=\\ =-{\text{Sh St}}_{1}\left(T_{c}-T_{g}\right)+Q_{comb}\pi_{2}W+\frac{\text{Sh}}{{\text{Pe}}_{1}}\left(1-a_{g}\right)\Delta T_{c}\\ -\;\rho_{cm}Q_{melt1}\frac{\partial F}{\partial t}-Q_{evap1}\frac{\partial {\rho^{\prime }}_{cm}}{\partial t}, \end{array}$$

(13) $$\begin{array}{l} \rho_{g}\left(\frac{\partial T_{g}}{\partial t}+\text{Sh }\left(\nu_{g}\cdot \nabla \right)\;T_{g}\right)=\text{Sh }{\text{St}}_{2}\left(T_{c}-T_{g}\right)+\frac{\text{Sh}}{{\text{Pe}}_{2}}a_{g}\Delta T_{g}\\ +\;Q_{evap2}\frac{\partial {\rho^{\prime }}_{gm}}{\partial t}, \end{array}$$

(14) $$\begin{array}{l} \;\rho_{g}\left(1+\chi \left(1-a_{g}\right)\right)\;\left(\frac{\partial \nu_{g}}{\partial t}+\text{Sh }\left(\nu_{g}\cdot \nabla \right)\;\nu_{g}\right)=\\ \;-\;a_{g}\text{Eu Sh}\nabla p-\frac{a_{g}^{2}\text{Sh}}{\text{Re }\pi }\nu_{g}\frac{T_{g}^{1.5}}{{\overset{⌢}{c}}_{s2}+T_{g}}-\\ -\frac{\text{Sh}}{\text{Fr}}\rho_{g}\frac{g}{\left|g\right|}-\left[\left(1-\mu_{p}\right)\pi_{2}\pi_{3}W+\pi_{3}\frac{\partial {\rho^{\prime }}_{cm}}{\partial t}-\frac{\partial {\rho^{\prime }}_{gm}}{\partial t}\right]\nu_{g}, \end{array}$$

(15) $$\begin{array}{l} \frac{\partial \rho_{g}}{\partial t}+\text{Sh }\nabla \cdot \left(\rho_{g}\nu_{g}\right)=\left(1-\mu_{p}\right)\pi_{2}\pi_{3}W+\pi_{3}\frac{\partial {\rho^{\prime }}_{cm}}{\partial t}\\ \;-\;\frac{\partial {\rho^{\prime }}_{gm}}{\partial t}{,}_{p}=\frac{\rho_{g}T_{g}}{a_{g}}, \end{array}$$

(16) $$\begin{array}{l} \rho_{g}\left(\frac{\partial C}{\partial t}+\text{Sh }\left(\nu_{g}\cdot \nabla \right)\;C\right)=\frac{\text{Sh}}{\text{Re Sc}}\nabla \cdot \left(\rho_{g}D_{g}\nabla C\right)\\ -\;\mu_{g}\pi_{2}\pi_{3}W- \end{array}$$

(17) $$\begin{array}{l} -\left[\left(1-\mu_{p}\right)\pi_{2}\pi_{3}W+\pi_{3}\frac{\partial {\rho^{\prime }}_{cm}}{\partial t}-\;\frac{\partial {\rho^{\prime }}_{cm}}{\partial t}\right]C,\\ W=\left(1-\eta \right)C\exp\;\left(-E/T_{c}\right),\frac{\partial \eta }{\partial t}=\pi_{2}W, \end{array}$$

(18) $$\frac{\partial \rho_{gm}}{\partial t}+\text{Sh}\nabla \cdot \left(\rho_{gm}\nu_{g}\right)=\pi_{3}\frac{\partial {\rho^{\prime }}_{cm}}{\partial t}-\;\frac{\partial {\rho^{\prime }}_{gm}}{\partial t},$$

(19) $$\rho_{cf}=1-\eta ,\rho_{cp}=\mu_{p}\eta ,\frac{\partial \rho_{cm}}{\partial t}=-\frac{\partial {\rho^{\prime }}_{cm}}{\partial t}+\frac{1}{\pi_{3}}\frac{\partial {\rho^{\prime }}_{gm}}{\partial t},$$

(20) $$\begin{array}{l} a_{g}=a_{g0}+\left(a_{cf0}-a_{cpEnd}\right)\eta +\left(\rho_{cm0}-\rho_{cm}\right)\pi_{6},\\ D_{g}=T_{g}^{b}. \end{array}$$

The boundary conditions for system (12)–(20) are the following. At the inlet of the reactor the gas velocity or the gas pressure is known, also the gas temperature, the mass concentration for the oxidizer and effective density of metal in gas are known. At the outlet of the reactor the gas pressure and conditions for gas temperature, oxidizer concentration and effective density of metal in gas are known. Also, we specify the conditions of heat exchange at the open boundaries and impermeable walls of the reactor. Thus, the boundary conditions for system of Equations (12)–(20) can be written in the following form:

(21) $$\begin{array}{l} \;p{|}_{x\in G_{1}}=p_{0}\left(x\right)or\;\nu_{g}{|}_{x\in G_{1}}=\nu_{g0}\left(x\right),\;T_{g}{|}_{x\in G_{1}}=T_{g0},\;C{|}_{x\in G_{1}}=C_{0}\\ \text{ and }\rho_{gm}{|}_{x\in G_{1}}=0,\text{if }\nu_{g}{|}_{x\in G_{1}}\cdot \;n{|}_{x\in G_{1}}\leq 0, \end{array}$$

(22) $$\begin{array}{l} \;p{|}_{x\in G_{1}}=p_{0}\left(x\right),\;\partial T_{g}/\partial n{|}_{x\in G_{1}}=0,\;\partial C/\partial n{|}_{x\in G_{1}}=\;0\\ \text{ and }\partial \rho_{gm}/\partial n{|}_{x\in G_{1}}=0,\text{if }\nu_{g}{|}_{x\in G_{1}}\cdot \;n{|}_{x\in G_{1}}>0, \end{array}$$

(23) $$\begin{array}{l} \;\partial T_{c}/\partial n{|}_{x\in G_{1}}=\text{Bi}\left(T_{g0}-\;T_{c}{|}_{x\in G_{1}}\right), \end{array}$$

(24) $$\begin{array}{l} \;\partial T_{c}/\partial n{|}_{x\in G_{2}}=0,\;\partial T_{g}/\partial n{|}_{x\in G_{2}}=0,\;\nu_{g}{|}_{x\in G_{2}}\cdot \;n{|}_{x\in G_{2}}=0. \end{array}$$

Here *G*_1_ is the object boundary opened to an atmosphere, *G*_2_ is the impermeable walls of the object, ***n*** is the outward vector directed normally to *G*_1_ or to *G*_2_; *C*_0_, *p*_0_, *T*_*g*0_ are the mass concentration of the oxidizer, the gas pressure and the gas temperature in the vicinity of the object, and Bi = $\frac{\beta H}{\lambda_{c}}$, where β is the heat removal coefficient.

In the calculation, Equation (12) splits into three equations, from which three functions are determined—*T*_*c*_, *F* ρ′_*cm*_–as follows:

- when *T*_*c*_ ≠ *T*_*melt*_ and *T*_*c*_ ≠ *T*_*evap*_, then ∂ρ′_*cm*_/∂*t* = 0, ∂*F*/∂*t* = 0, and we can determine *T*_*c*_,
- when *T*_*c*_ = *T*_*melt*_, then ∂*T*_*c*_/∂*t* = 0 and ∂ρ′_*cm*_/∂*t* = 0, and we can determine *F*;
- when *T*_*c*_ = *T*_*evap*_, then ∂*T*_*c*_/∂*t* = 0 and ∂*F*/∂*t* = 0, and we can determine ρ′_*cm*_.

Equation (13) splits into two equations, from which two functions are determined—*T*_*g*_ and ρ′_*gm*_–as follows:

- when *T*_*g*_ ≠ *T*_*evap*_, then ∂ρ′_*gm*_/∂*t* = 0, and we can determine *T*_*g*_,
- when *T*_*g*_ = *T*_*evap*_, then ∂*T*_*g*_/∂*t* = 0, and we can determine ρ′_*gm*_.

Note that the system of Equations (12)–(20) with boundary conditions (21)–(24) is based on two models previously developed and tested: model of heterogeneous combustion of porous media (Levin and Lutsenko, 2017; Lutsenko, 2018) and model of gas flow through granular materials with phase changes (Levin et al., 2018; Lutsenko and Fetsov, 2019). The computational algorithms used for these models have formed the basis of the numerical method for solving the system of Equations (12)–(20). According to the method the energy equations, momentum conservation equation, equation for oxidizer concentration and equation of mass conservation for evaporated metal in gas are transformed into explicit finite difference equations. The condensed phase temperature, the liquid fraction in the metal, the decrease in the effective density of the condensed metal due to evaporation, the gas temperature, the decrease in the effective density of the evaporated metal due to condensation, the gas filtration velocity, the oxidizer concentration and the effective density of metal in gas phase are determined from these equations. The continuity equation is transformed into implicit finite difference equation, from which the gas pressure is determined using Thomas algorithm (Tannehill et al., 1997) and taking into account the perfect gas equation of state. The effective gas density and the remaining unknown quantities are determined trivially from the perfect gas equation of state and other closure equations.

## Results and Discussion

To study the mass transfer of metal compounds under conditions of filtration combustion, we consider a cylindrical reactor with a porous medium consisting of a mixture of particles of birch coal (fuel), chamotte bricks (inert material) and cadmium (metal). Thus, the characteristics of a combustible, inert material and metal were assumed to be equal to the characteristics of birch coal, chamotte and cadmium, respectively. It should be noted that the selected characteristics of the materials are close to those of coal gasification in the works of other researchers (Aldushin, 1997; Rozenberg et al., 2009; Manelis et al., 2016; Antonov et al., 2017). We suppose that the solid phase is premixed, its properties at each point in the reactor at the initial time are constant and equal. To carry out combustion, air is supplied into the reactor; the flow in the reactor is assumed to be one-dimensional. At the time of ignition, the pressure at the object bottom rapidly increases up to *p*_01_and remains constant; the pressure at the object top *p*_*h*_ does not change. To initiate the combustion, the temperature of the solid phase in ignition zone, which is located near the inlet of the reactor and has a width of *H*_1_, becomes instantly equal to *T*_*c*0_ that is much higher than the initial temperature. After ignition of the fuel, the combustion wave passes through the reactor. The initial characteristics of the system are presented in the Table 1.

**Table 1:** The initial characteristics of the system.

**Parameter**	**Value**	**Parameter**	**Value**	**Parameter**	**Value**
*H*	1 m	β	20 J/(m^2^ K s)	*k*	1.6 × 10^4^ 1/s
*a*_*cf*0_	0.1	λ_*c*_	1.1 J/(m K s)	*E*	9.8 × 10^4^ J/mol
ρ_*cf*0_	*a*_*cf*0_ * 250 kg/m^3^	λ_*g*_	0.034 J/(m K s)	*D_*g*0_*	1.82 × 10^−5^ m^2^/s
*a*_*cm*0_	0.01; 0.001	*c*_*S*1_	1.458 × 10^−6^ kg/(m s K^1/2^)	*b*	1.724
ρ_*cm*0_	*a*_*cm*0_ * 8,650 kg/m^3^	*c*_*S*2_	110.4 K	*μ_*g*_*	2.667
*a*_*g*0_	0.3	*k_1_*	10^−8^ m^2^	*T_*g*0_*	300 K
*a*_*ci*0_	1−*a*_*cf*0_−*a*_*cm*0_−*a*_*g*0_	χ	0.5	*C_0_*	0.23
ρ_*ci*0_	*a*_*ci*0_*1, 300 kg/m^3^	*g*	9.8 m/s^2^	*T_*c*0_*	900 K
*c*_*g*_	1,000 J/(kg K)	*R*	8.31441 J/(mol K)	*T_*melt*_*	594 K
*c*_*cf*_	1,200 J/(kg K)	*M*	2.993 × 10^−2^ kg/mol	*T_*evap*_*	1,038 K
*c*_*ci*_	1,400 J/(kg K)	*Q_*comb*_*	3.28 × 10^7^ J/kg	*p_01_*	(1.005; 1.01; 1.02) × 10^5^ Pa
*c*_*cm*_	264 J/(kg K)	*Q_*melt*_*	5.393 × 10^4^ J/kg	*p_*h*_*	1.00 × 10^5^ Pa
α	10^4^ J/(m^3^ K s)	*Q_*evap*_*	5.216 × 10^5^ J/kg	*H_1_*	0.1 m

**Figure 2:**
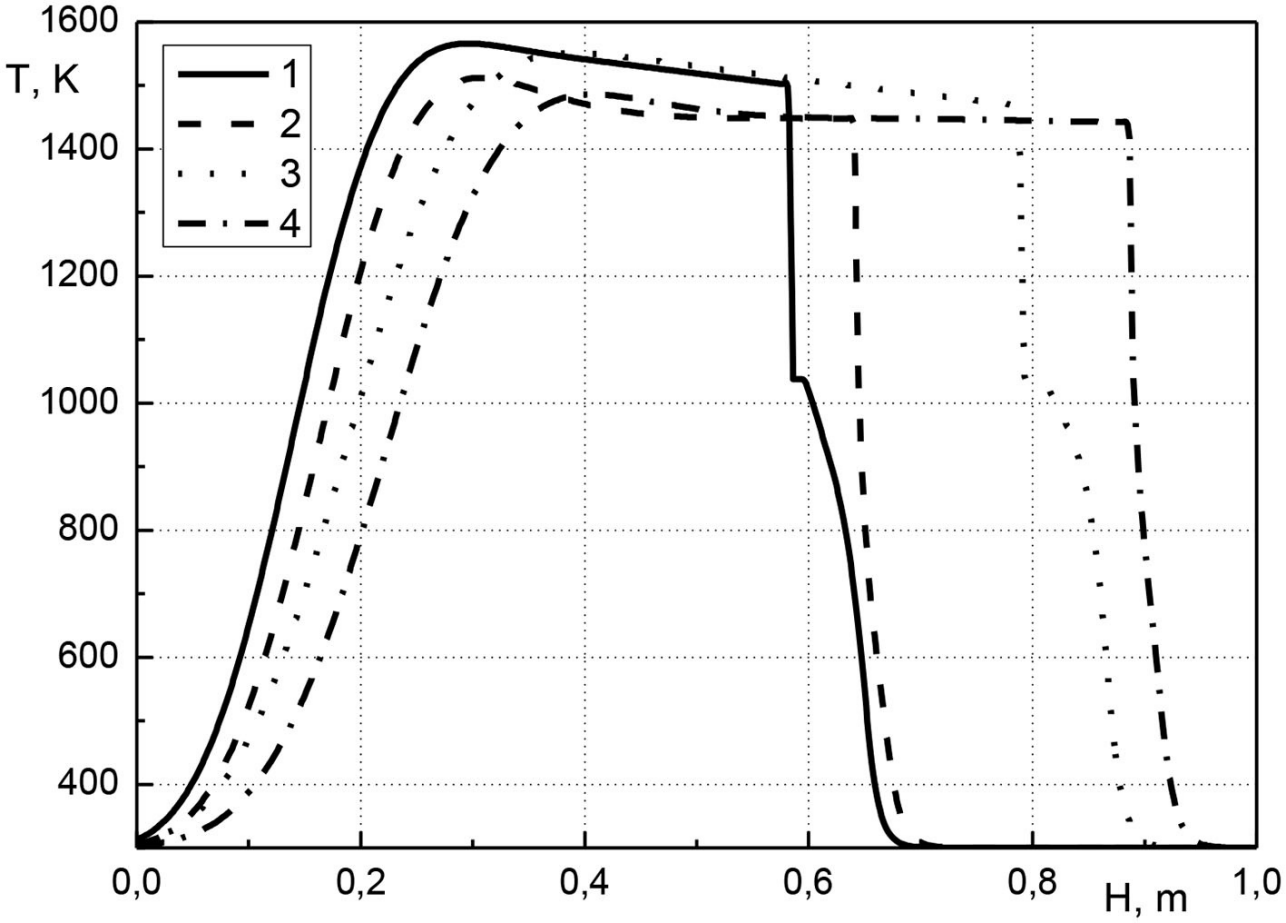
Condensed phase temperature profiles at various points in time. 1–*t* = 2,700 s, *a*_*cm*0_ = 0.01; 2–*t* = 2,700 s, *a*_*cm*0_ = 0.001; 3–*t* = 4,500 s, *a*_*cm*0_ = 0.01; 4–*t* = 4,500 s, *a*_*cm*0_ = 0.001.

In Figure 2 the distribution of the temperature of the condensed phase along the length of the reactor is shown at various points in time. The initial volumetric concentration of cadmium is equal to 0.001 or 0.01, the pressure at the inlet of the reactor is equal to 1.005 atm. Hereinafter, the presented calculations results were obtained using a uniform grid with mesh size 1/800. As seen from the figure, the combustion front moves through the reactor from its inlet to outlet. The maximum temperatures are close to 1,500 K. It can be seen that for same compositions (curves 1, 3 or 2, 4) the temperature profiles practically coincide, which indicates the achievement of a quasistationary state. Before the combustion front, there is the zone of cadmium evaporation. The zone of evaporation of the metal into gas expands (curves 1 and 3), which indicates the accumulation of cadmium before the combustion front. The initial cadmium concentration of 0.001 has almost no effect on the type of temperature profiles. Such a low metal concentration makes a negligible contribution to the thermal state of the system. An increase in cadmium concentration leads to a decrease in the velocity of the combustion front (curves 1, 2 or 3, 4). This can be explained by the fact that the higher the initial cadmium concentration, the more vaporized cadmium is transferred to the zone in front of the combustion wave, and this increases the temperature in this zone. The higher temperature in front of the combustion wave, the higher temperature in the combustion wave. At a constant gas pressure at the reactor inlet, increase in temperature of porous medium leads to a decrease in the gas velocity, that leads to a decrease in the velocity of the combustion wave. The evaporation of the metal into the gas phase makes it more difficult to filter the gas through the porous medium.

**Figure 3:**
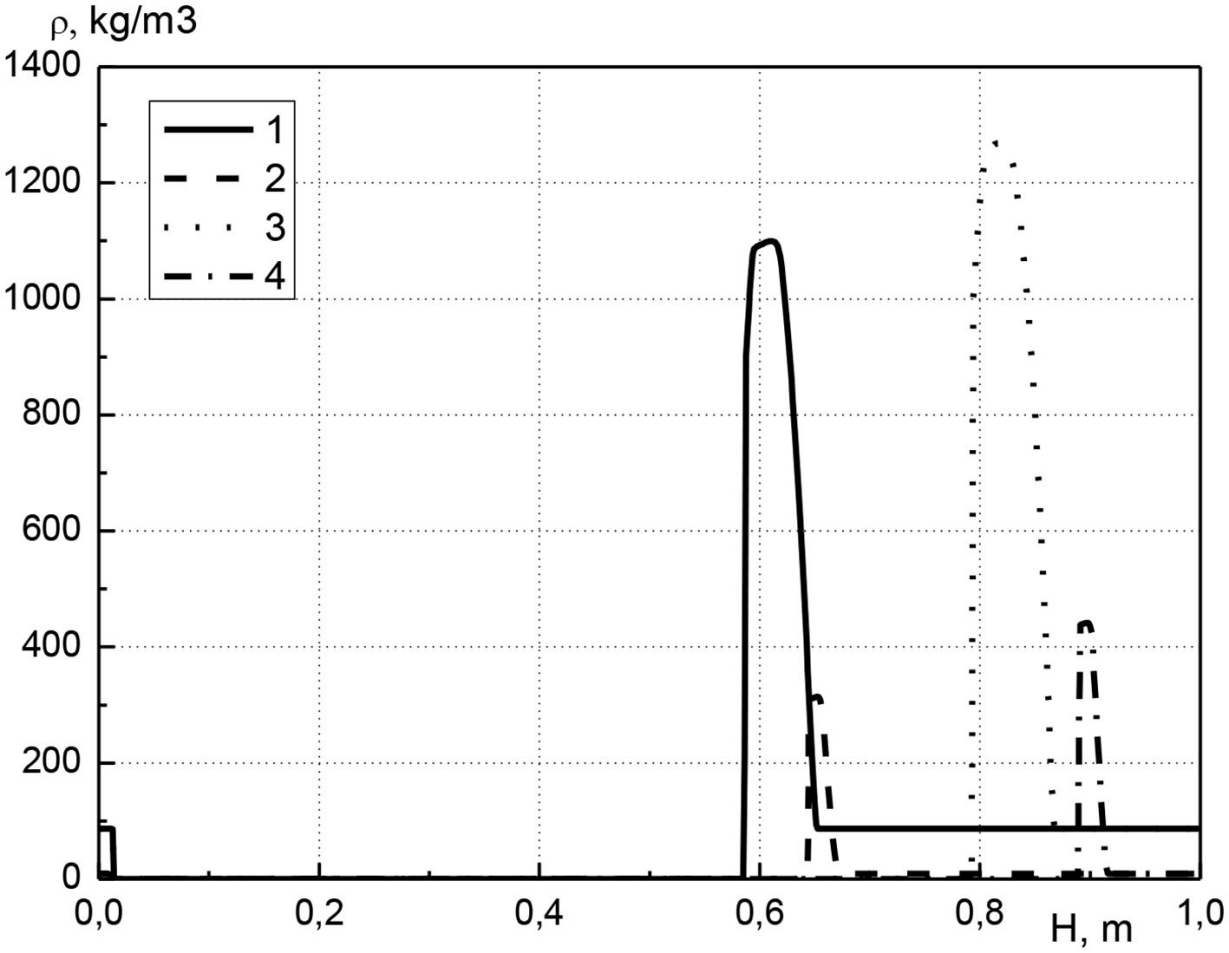
Profiles of condensed cadmium effective density at various points in time. 1–*t* = 2,700 s, *a*_*cm*0_ = 0.01; 2–*t* = 2,700 s, *a*_*cm*0_ = 0.001; 3–*t* = 4,500 s, *a*_*cm*0_ = 0.01; 4–*t* = 4,500 s, *a*_*cm*0_ = 0.001.

In Figure 3 the distribution of condensed cadmium effective density along the length of the reactor is shown at various points in time. The calculations are performed for the same parameters as in Figure 2. It should be noted that the effective density of substance is equal to the product of its volume concentration and true density, therefore, changes in the effective density of the condensed cadmium and its concentration are equivalent. The distribution of metal along the length of the reactor is as follows. At the beginning of the reactor, the cadmium concentration is zero, i.e., the metal is missing. Next is a narrow zone with a high metal content, which is located directly before the combustion front (see Figure 2). After this zone, the cadmium concentration is constant until the end of the reactor and is equal to the initial value. Over time, the maximum metal concentration increases (curves 1, 3 or 2, 4). It can be noted that the width of the zone with a high metal content remains almost constant (curves 1, 3 or 2, 4). Thus, we can conclude that the metal is transported through the reactor due to the continuous evaporation and condensation of the metal before the combustion front.

**Figure 4:**
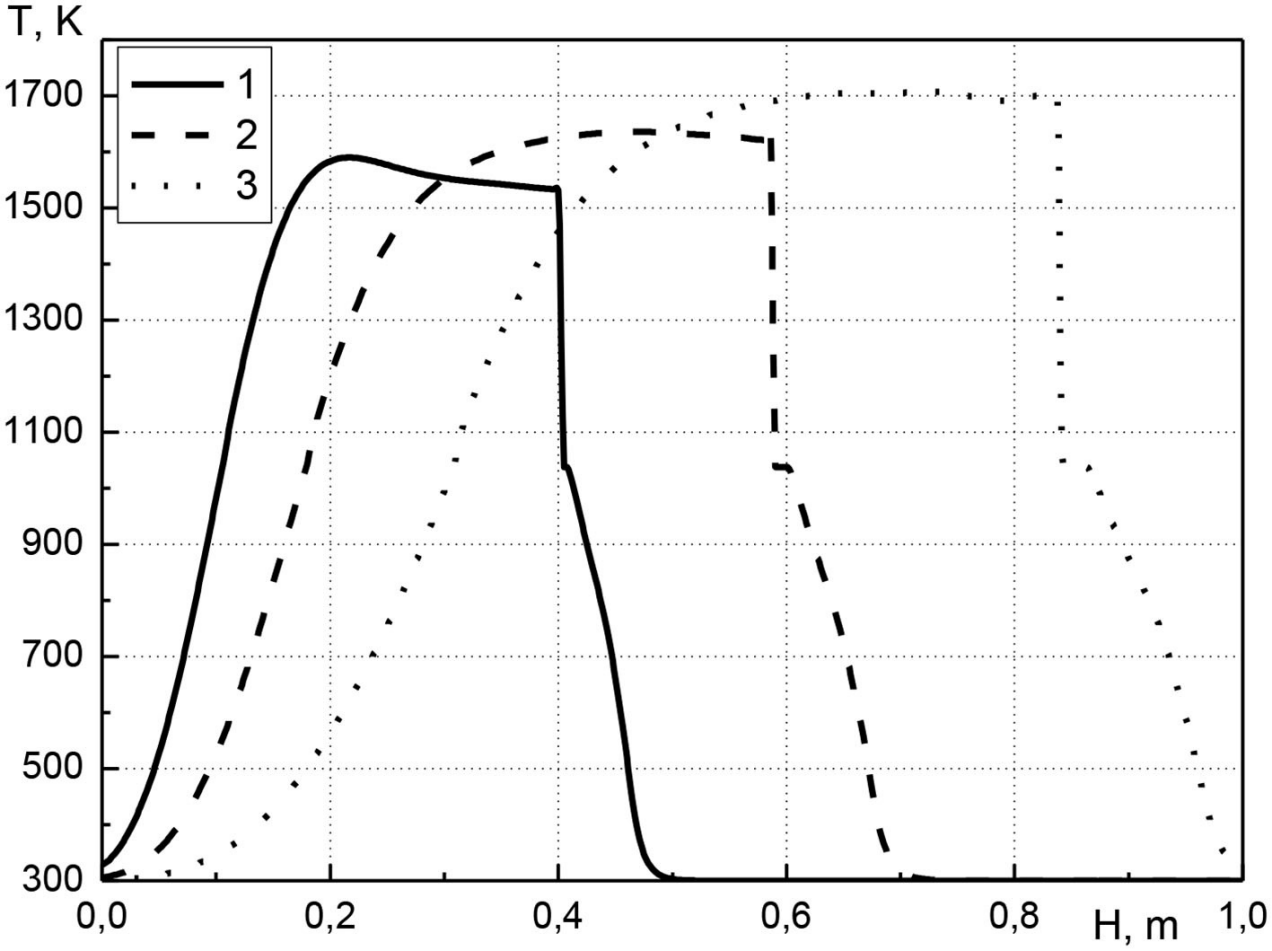
Condensed phase temperature profiles at time *t* = 1,500 s. 1–*p*_01_ = 1.005 atm; 2–*p*_01_ = 1.01 atm; 3–*p*_01_ = 1.02 atm.

In Figure 4 the distribution of the temperature of the condensed phase along the length of the reactor is shown for various pressure values at the inlet of the reactor for a point in time of 1,500 s from the start of the process. The pressure at the inlet of the reactor ranges from 1.005 to 1.02 atm; the initial volume concentration of cadmium is equal to 0.01. It can be seen that an increase in pressure at the inlet of the reactor leads to an increase in the maximum temperature. The maximum temperature value is realized in the combustion front where the main heat release occurs (curves 2 and 3). For curve 1, this is not observed due to the influence of the ignition stage. At the beginning of the process, the mixture ignites due to the heating of a part of the solid phase. In this case, a sharp rise in temperature occurs both due to the heat release of chemical reactions of fuel oxidation, and due to the preliminary heating of the solid phase. For the first case (curve 1), as the combustion front moves, the temperature in the front decreases slightly, and the temperature profile has convex. Over time, this convexity of the profile decreases due to thermal conductivity (see Figure 2, curves 2 and 4). An increase in pressure at the inlet of the reactor leads to an increase in gas flow and, as a consequence, to an increase in the velocity of the combustion front. With increasing the inlet gas pressure, an increase in the width of transition zone of the metal into the gas phase is observed, which indicates the accumulation of metal before the combustion front.

**Figure 5:**
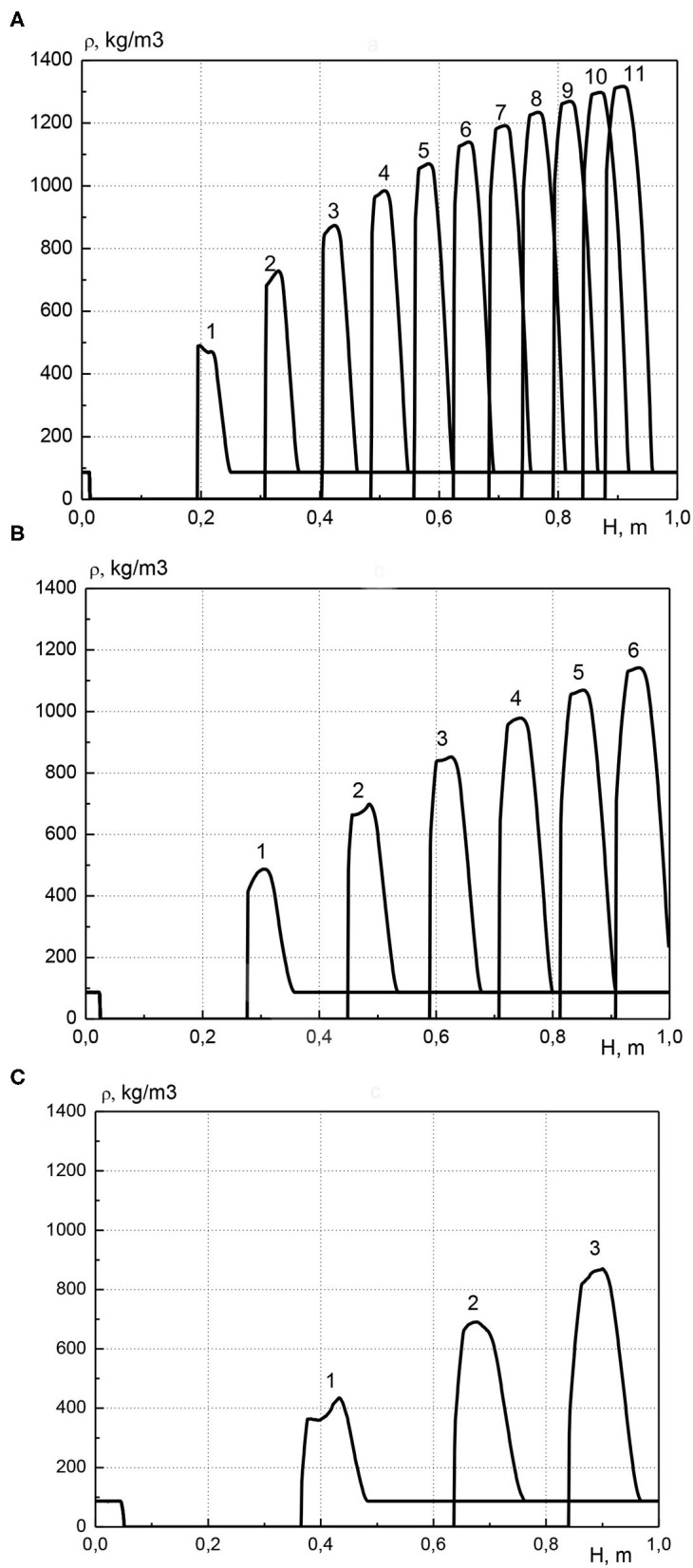
Profiles of condensed cadmium effective density at various points in time, curve number corresponds to time *t* = (curve number) × 500 s. **(A)** *p*_01_ = 1.005 atm; **(B)** *p*_01_ = 1.01 atm; **(C)** *p*_01_ = 1.02 atm.

In Figure 5 the distribution of condensed cadmium effective density along the length of the reactor is shown for various pressure values at the inlet to the reactor. Profiles are presented at various points in time in increments of 500 s. The calculations were performed for the same parameters as in Figure 4. For each pressure value at the inlet of the reactor, metal is transferred along the length of the reactor and accumulates before the combustion front. Moreover, as in Figure 3, the maximum concentration of the metal increases over time, and the width of the high metal content zone remains almost constant. When the pressure values at the inlet of the reactor is equal to 1.005, 1.01, and 1.02 atm, the maximal achievable concentration of cadmium is about 1,350, 1,150, and 900 kg/m^3^, respectively. The increase in pressure at the inlet of the reactor leads to the fact that the zone of high cadmium content decreases in height and increases in width, that is, the accumulation of metal occurs in a wider area. This is due to an increase in the velocity of the gas phase with increasing the gas pressure at the inlet of the reactor. Due to the increase in gas velocity, a particle of the evaporated metal, moving into an area with a low temperature, flies a greater distance until it completely condensed, this leads to the expansion of the metal-containing zone.

## Conclusions

To study the mass transfer of metal compounds, a model of filtration combustion of metal-containing combustible mixtures is developed. A system of dimensionless equations is obtained, and a solution method is briefly described. Using cadmium-containing mixture as an example, the main characteristics of filtration combustion are determined when the gas pressure at the reactor inlet is constant. It is shown that under the conditions of a filtration combustion wave, cadmium can evaporate into the gas phase and be transferred with gas through the reactor. Due to the evaporation and condensation of cadmium, it is transported and accumulated before the combustion front. It is shown that an increase in the initial concentration of the metal, as well as its accumulation before the combustion front, can affect the temperature profile. At a high initial concentration of cadmium, a zone before the combustion front is substantially heated due to the condensation of the evaporated metal, and the condensed metal begins to evaporate in this zone. As a result, the zone expanding with time with a constant temperature equal to the phase transition temperature is clearly visible on the profile of the condensed medium temperature. An increase in pressure at the inlet of the reactor leads to an increase in the maximum temperature and velocity of the combustion front, due to an increase in gas flow. The increase in pressure at the inlet of the reactor leads to the fact that the high cadmium content zone decreases in height and increases in width, that is, the accumulation of metal occurs in a wider area. It is shown that a 4-fold increase in the pressure difference at the open boundaries of the reactor leads to a decrease in the maximum metal concentration by about 1.5 times. The possibility of controlling the mass transfer of metal compounds under the conditions of a filtration combustion wave with the aim of concentrating them is shown. An increase in the concentration of metals due to mass transfer will subsequently make it economically feasible to extract them by traditional methods.

## Data Availability Statement

The datasets generated for this study are available on request to the corresponding author.

## Author Contributions

ES and NL: substantial contributions to the design of the work and model investigated and resolved. MT: analysis and interpretation of data for the work and provide approval for publication of the content. All authors contributed to the article and approved the submitted version.

## Conflict of Interest

The authors declare that the research was conducted in the absence of any commercial or financial relationships that could be construed as a potential conflict of interest. The handling editor is currently organizing a Research Topic with one of the authors MT.
